# Increased Frequency of CD4 and CD8 Regulatory T Cells in Individuals under 15 Years with Multibacillary Leprosy

**DOI:** 10.1371/journal.pone.0079072

**Published:** 2013-11-14

**Authors:** Camila Fernandes, Heitor Sá Gonçalves, Paula Brito Cabral, Helena Câmara Pinto, Maria Isabel Moraes Pinto, Lilia Maria Carneiro Câmara

**Affiliations:** 1 Department of Pathology and Legal Medicine, Medical Laboratory Immunology, Federal University of Ceará, Fortaleza, Ceará, Brazil; 2 Dermatology Center Dona Libânia, Fortaleza, Ceará, Brazil; 3 Walter Cantídio University Hospital, Fortaleza, Ceará, Brazil; 4 University of Fortaleza, Fortaleza, Ceará, Brazil; 5 Department of Pediatrics, Federal University of São Paulo, São Paulo, São Paulo, Brazil; National Institute of Infectious Diseases, Japan

## Abstract

**Background:**

Leprosy is a chronic disease, caused by *Mycobacterium leprae,* which poses a serious public health problem worldwide. Its high incidence in people under 15 years old in Ceará state, Brazil, reflects the difficulty of its control. The spectrum of clinical manifestations is associated with the immune response developed, with the Th1 and Th2 responses being related to the paucibacillary and multibacillary forms, respectively. Regulatory T cells (Treg), which can suppress Th1 and Th2 response, have received special attention in the literature and have been associated with development of chronic infections. However, their role in leprosy in individuals under 15 years old has not yet been elucidated. We evaluated the frequency of CD4^+^/CD8^+^CD25^high^FOXP3^+^ and CD4^+^/CD8^+^CD25^high^FOXP3^high^ cells in leprosy patients and household contacts, in both cases under 15 years old.

**Methodology/Principal Findings:**

PBMC from 12 patients and 17 contacts were cultured for 72 hours with anti-CD3 and anti-CD28 (activators) or with activators associated with total sonicated fraction of *M. leprae*. After culture, the frequency of CD4^+^/CD8^+^ Treg was identified by flow cytometry. Cells stimulated by activators and antigen from multibacillary patients showed Treg frequencies almost two times that of the contacts: CD4^+^FOXP3^+^ (21.93±8.43 vs. 13.79±8.19%, p = 0.0500), CD4^+^FOXP3^high^ (10.33±5.69 vs. 5.57±4.03%, p = 0.0362), CD8^+^FOXP3^+^ (13.88±9.19 vs. 6.18±5.56%, p = 0.0230) and CD8^+^FOXP3^high^ (5.36±4.17 vs. 2.23±2.68%, p = 0.0461). Furthermore, the mean fluorescence intensity of FOXP3 in Treg was higher in multibacillary patients than in the contacts. Interestingly, there was a positive correlation of the bacillary index and number of lesions with the frequency of all Treg evaluated in patients.

**Conclusions/Significance:**

We have demonstrated for the first time that multibacillary leprosy patients under 15 years old have greater CD4^+^ and CD8^+^ Treg frequencies and these correlate with clinical and laboratorial aspects of disease. These findings suggest the involvement of these cells in the perpetuation of *M. leprae* infection.

## Introduction

Leprosy is a chronic disease caused by *Mycobacterium leprae*. It is characterized by skin lesions with changes in sensitivity and neural damage, with possible incapacitation and deformities. The disease is an important public health problem worldwide [1], especially in Ceará state, in Brazil, where it is endemic. This state occupies the 13th place in Brazil and 4th place in the Northeast Brazilian region in new case detection rate for leprosy [2].

The disease’s incidence among individuals under 15 years old is an important epidemiological indicator, because it is directly related to active transmission foci in the community [3]. The new case detection rate in this age group in Ceará state is high, 5.3 cases/100,000 inhabitants in 2012. In addition to the high number of cases, delayed diagnosis in the region influences the high number of people with disabilities and deformities resulting from the disease [2].

The household contacts (HHC) of leprosy patients have a high risk of acquiring the disease, which is influenced by the contact age and gender, presence of a BCG vaccination scar and the clinical form of the index case. Among children and adolescents, this risk increases in the age range of 10 to 19 years [4].

For treatment purposes, the Brazilian Ministry of Health and the World Health Organization classify leprosy into two forms: paucibacillary (PB) and multibacillary (MB), according to the number of lesions, degree of neural compromise and/or bacilloscopy result. The PB form is characterized by the presence of up to five characteristic lesions, negative bacilloscopy and no nerve affected, whereas the MB form has more than five lesions and/or positive bacilloscopy and/or at least one nerve trunk compromised [5].

The majority of individuals infected by *M. leprae* do not get sick, with only a small portion developing clinical manifestations [6]. This makes the disease even more intriguing, and raises questions about the factors responsible for the greater susceptibility of some people. The balance between Th1 and Th2 responses was for a long time utilized to explain the clinical forms of the disease and the susceptibility to getting sick, since the spectrum of clinical manifestations is related to the immunological response pattern of the host. The cell-mediated immune response characterizes the PB form, with a predominance of Th1 cytokines, limitation of bacillary proliferation and relative resistance to the pathogen, while the lack of Th1 response and predominance of Th2 response characterizes the MB form, which has intense bacillary multiplication [7].

However, the development of the immune response, which controls the growth of the pathogen and limits the tissue lesion caused by an exacerbated response, is important for the resolution of the infection [8]. The response mediated by regulatory T cells (Treg) has been related to infection susceptibility [9], since it can promote the growth and persistence of the pathogens [10]. Hence, these cells have gained importance in understanding the immunopathogenesis of leprosy, bringing into question the paradigm of Th1 and Th2 response, as suggested by Kumar et al. by analyzing the profile of various genes in leprosy patients. In multibacillary patients they found down-regulation of genes related to Th1 and Th2 immune response and up-regulation of those related to Th3 immune response, which is mediated by Treg cells, with increased levels of TGF-β as a favorable factor for growth and survival of *M.* *leprae* [11].

Treg comprise about 1–2% of CD4^+^ T lymphocytes in human peripheral blood [12] and can suppress a wide range of immune cells, being important for maintaining homeostasis [13]. In humans they are heterogeneous populations that can be characterized by expression of FOXP3 (Forkhead box P3) and by high expression of CD25 (Interleukin-2 receptor α-chain) [14]. The CD4^+^CD25^high^FOXP3^+^ Treg population has been more extensively studied, but according to Miyara et al., this conventional subset of Treg can be divided into different fractions according to the expression of FOXP3. In human peripheral blood, the fraction CD4^+^CD25^high^FOXP3^high^ is composed of activated Treg, which have higher suppressor capacity *in vitro* [15]. In fact, expression of *Foxp3*, a main control gene of Treg responsible for their development and function, is critical for its regulatory function [14].

Although CD8^+^ Treg have received less attention in the literature [16], the existence of CD8^+^ Treg is now well established, which can expand after stimulation with different antigens [17]. CD8^+^ Treg, isolated from healthy humans, express CD25 and FOXP3, produce IL-10 and are functionally suppressive when stimulated *in vitro* [18]. CD8^+^CD25^high^FOXP3^+^ cells also have been investigated in humans and demonstrated suppressive capacity *in vitro* [19]. In leprosy, the first cells with suppressive activity were characterized by the expression of the CD8 molecule [20], [21] and were associated with hyporesponsiveness in lepromatous patients [22], but the CD8^+^ Treg frequency and their relation to leprosy’s clinical forms has not yet been demonstrated. Although the role of Treg in *M. leprae* infection is not clearly elucidated, the importance of these cells in the perpetuation of chronic infections has been demonstrated in infections by other intracellular microorganisms. In mice infected with *Mycobacterium tuberculosis* antigen-specific CD4^+^CD25^+^ Treg were capable of preventing the expansion and mobilization of effector T cells to the infection site, promoting the survival of the microorganism [23]. Hisaeda et al. demonstrated that the depletion of CD25^+^ cells protects mice from death due to infection by the lethal strain of *Plasmodium yoelii* [24]. The depletion of Treg reduces the bacterial load in lungs of mice infected with *Mycobacterium tuberculosis* [25].

In this study, we evaluated for the first time the frequency of CD4^+^ and CD8^+^ Treg in leprosy patients and HHC, in both cases under 15 years old, and their relation with the clinical forms of the disease, to shed light on their role in the pathogenesis of the disease in this age group. Studies in this age group can help identify factors that influence the development of the disease and can be used in early diagnosis.

## Methods

### Ethics Statement

This study was approved by the ethics committee of Federal University of Ceará under protocol number 161/11. All legal guardians signed the informed consent form.

### Characterization of Patients and HHC

Patients and HHC were seen in the period of March to June of 2012, in the Centro de Dermatologia Sanitária Dona Libânia, a reference center for the diagnosis and treatment of leprosy in Ceará state, Brazil. HHC were defined as those who lived with the index case at diagnosis. All participants had been vaccinated at birth with BCG, evidenced by the presence of the vaccination scar and/or vaccination registration card from the Ministry of Health. A dermatological-neurological examination of HHC was carried out by professionals of the reference center to rule out the presence of the disease. None of patients and HHC were on immunosuppressive therapy or had associated comorbidities. The patients included had clinical and laboratory diagnosis of leprosy and we excluded those with type 1 or 2 reaction. After authorization from the legal guardian and signed informed consent, a questionnaire was filled out to obtain information regarding identification, BCG vaccination, physical examination and laboratory tests of the patient or HHC and of their index case, and 10 mL of peripheral venous blood was collected by a trained professional. The blood sample for peripheral blood mononuclear cell (PBMC) culture and flow cytometry was transported refrigerated within 4 h of drawing the blood sample.

Twenty-nine individuals were evaluated, including 12 untreated patients and 17 HHC. Among the 12 patients who participated in the study, there were 5 females and 7 males, and the median age was 12 years (range 5 to 15 years). Six patients had the MB form and the other 6 had the PB form. Among the 17 HHC, there were 11 males and 6 females, with a median age of 5 years (range 1 to 14 years), and 8 individuals were contacts of MB patients and 9 of PB patients. The median time of treatment of the index case in the HHC group was 14 days (range 1 to 37 days). The contacts had adults as index cases, with the exception of two who were contacts of two individuals under 15 years who participated in the survey.

### Stimulation of PBMC

The culture of PBMC was adapted from that described by Spencer et al. [26]. PBMC were obtained by separation in a Ficoll gradient (GE Healthcare, USA), and the concentration was adjusted to 2.5×10^6^ cells/mL in AIM-V culture medium (GIBCO, USA). PBMC samples from patients and contacts were stimulated with anti-CD3 antibodies (UCHT1, BD Biosciences, USA) and anti-CD28 antibodies (CD28.2, BD Biosciences) [27], both soluble and at a concentration of 0.5 µg/mL, or with anti-CD3 and anti-CD28 antibodies combined with the total sonicated fraction of *M. leprae* (MLT), at a concentration of 20 µg/mL. The cell suspension was distributed, in duplicate, in a 96-well plate (BD Biosciences) and cultivated in a 5% CO_2_ humidified atmosphere for 72 h at 37°C.

### Reagents for Characterization of Treg

The anti-FOXP3 PE (259D/C7, BD Biosciences) was chosen because of the good separation between FOXP3^−^ and FOXP3^+^ cells and lower nonspecific binding [28], besides its good stability in the detection of FOXP3 in cells stimulated with anti-CD3 and anti-CD28 after several days of culture [29]. The antibodies anti-CD25 FITC (M-A251, BD Biosciences), anti-CD4 PerCP-Cy5.5 (RPA-T4, Ebioscience, USA), anti-CD8 PerCP-Cy5.5 (RPA-T8, Ebioscience), anti-CD8 APC (RPA-T8, BD Biosciences) and anti-CD3 APC (OKT.3, BioLegend or Ebiosciences, USA) were titered, as well as anti-FOXP3, where the best concentration was chosen based on the highest percentage of positive cells and mean fluorescence intensity (MFI). The human FOXP3 buffer set from BD Biosciences was used for fixation and permeabilization.

### Preparation of Cells for Flow Cytometry

After culture, 2 mM of ethylenediamine tetraacetic acid was added to dislodge the adherent cells. The cells were labeled with surface antibodies for 30 min at 21°C in the dark. Intracellular labeling of FOXP3 was performed according to the specifications of the manufacturer. The cells were fixed in 1% paraformaldehyde and stored at 4°C. Flow cytometry was carried out within 24 h of cell preparation.

### Data Acquisition and Analyses

The data were collected using a FACSCalibur flow cytometer (BD Biosciences), with measurement of up to 400,000 events per sample in the lymphocyte gate. The results were analyzed with the FlowJo program (version 7.6.5; Treestar US, Ashland, OR).

### Statistical Analysis

The data were analyzed using the GraphPad Prism version 5 statistical program. The unpaired two-tailed t-test was used to determine differences between two variables and Pearson’s test was used for correlation analyses. Results were expressed as mean and standard deviation and *p*≤0.05 was considered significant.

## Results

### Gating Strategy for Identification of Treg

**Figure 1:**
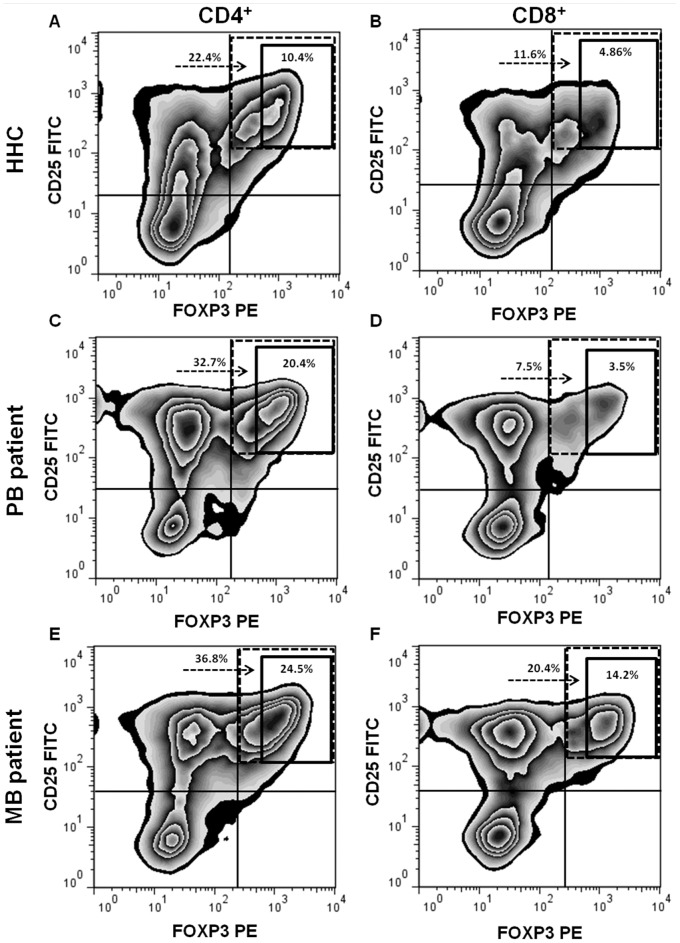
Representative FACS plots of CD4^+^/CD8^+^ Treg frequencies in contacts and leprosy patients. The frequency of Treg in CD4^+^ (A, C and E) or CD8^+^ (B, D and F) lymphocyte gate after 72-hour culture of peripheral blood mononuclear cells with anti-CD3 and anti-CD28 associated with total sonicated fraction of *M. leprae.* Identification of CD4^+^/CD8^+^CD25^high^FOXP3^+^ (dotted line) and CD4^+^/CD8^+^CD25^high^FOXP3^high^ (solid line) cells in household contact (HHC) of leprosy patient (A and B), paucibacillary (PB) patient (C and D) and multibacillary (MB) patient (E and F). Numbers represent percentages of events within each Treg gate in the total CD4^+^/CD8^+^ lymphocyte population.

Representative FACS plots of CD4^+^ and CD8^+^ Treg frequencies in HHC, PB and MB patients are shown in Figure 1. Positivity for CD25 and for FOXP3 was defined in each individual based on isotypic controls. CD25^high^ was defined by fluorescence intensity above 10^2^. Among the CD25^high^FOXP3^+^ population, we observed a distinct population of FOXP3^high^ cells, which we defined as CD25^high^FOXP3^high^ cells. We adjusted the gate of the FOXP3^high^ cells for each individual and the cut-off for FOXP3^high^ intensity ranged from 3.10^2^ to 7.10^2^. The results of anti-CD3, anti-CD28 and MLT stimulated cells showed the same differences between groups that were observed with activators only. We decided to show the results that include the specific stimulus of *M. leprae*.

### Treg Frequency Differs between Patients and Contacts

**Figure 2:**
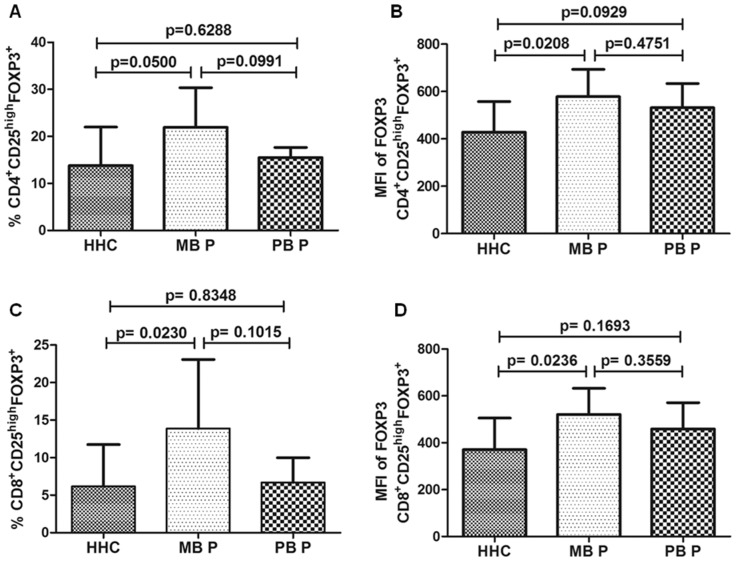
The frequency of CD4^+^/CD8^+^CD25^high^FOXP3^+^ cells and FOXP3 mean fluorescence intensity (MFI) in leprosy patients and contacts. The frequency of Treg in CD4^+^ or CD8^+^ lymphocyte gate after 72-hour culture of peripheral blood mononuclear cells with anti-CD3 and anti-CD28 associated with total sonicated fraction of *M. leprae.* Comparison between household contacts (HHC), multibacillary patients (MB P) and paucibacillary patients (PB P), with regard to frequency of CD4^+^CD25^high^FOXP3^+^ (A) and CD8^+^CD25^high^FOXP3^+^ (C) cells and to FOXP3 MFI in CD4^+^CD25^high^FOXP3^+^ (B) and CD8^+^CD25^high^FOXP3^+^ (D) cells. Unpaired two-tailed Student t-test, *p*≤0.05 was considered significant. Results are presented as mean with SD.

Initially, we evaluated CD4^+^ and CD8^+^CD25^high^FOXP3^+^ Treg frequencies. There were no differences in these Treg frequencies in leprosy patients versus HHC (CD4^+^ Treg, 18.70±6.77 vs. 13.79±8.19%, p = 0.0996 and CD8^+^ Treg, 10.29±7.57 vs. 6.18±5.56%, p = 0.1033, data not shown). When patients were separated into MB and PB, the MB patients showed a greater frequency of Treg than HHC: CD4^+^CD25^high^FOXP3^+^21.93±8.43 vs. 13.79±8.19% (Figure 2A) and CD8^+^CD25^high^FOXP3^+^13.88±9.17 vs. 6.18±5.56% (Figure 2C). There were no differences between PB patients and HHC in Treg frequency (Figures 2A and C). In addition, patients had FOXP3 MFI in Treg higher than did HHC (CD4^+^ Treg, 554.8±106.7 vs. 428±129.9, p = 0.0098 and CD8^+^ Treg, 490±111.2 vs. 371.2±134.7, p = 0.0184, data not shown). These differences in FOXP3 MFI were significant only between MB patients and HHC (CD4^+^ Treg, 578.2±115.3 vs. 428±129.9, Figure 2B; and CD8^+^ Treg, 521.2±110.9 vs. 371.2±134.7, Figure 2D).

**Figure 3:**
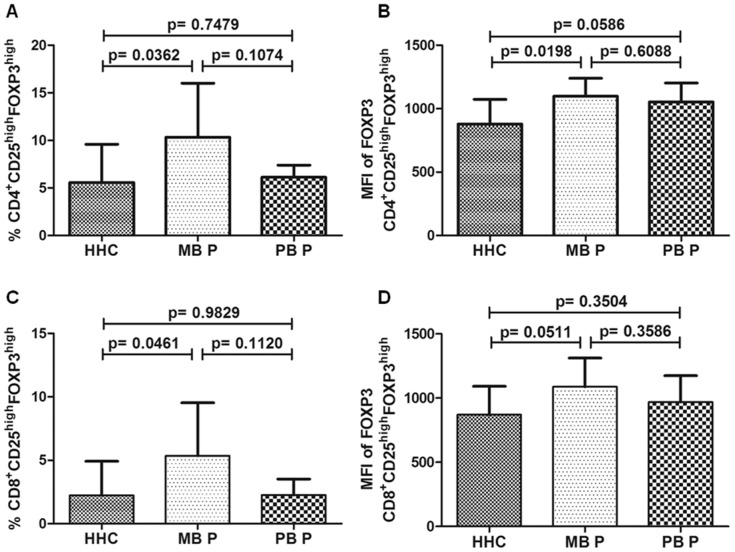
CD4^+^/CD8^+^CD25^high^FOXP3^high^ cell frequency and FOXP3 mean fluorescence intensity (MFI) in leprosy patients and contacts. The frequency of Treg in CD4^+^ or CD8^+^ lymphocyte gate after 72-hour culture of peripheral blood mononuclear cells with anti-CD3 and anti-CD28 associated with total sonicated fraction of *M. leprae.* Comparison between household contacts (HHC), multibacillary patients (MB P) and paucibacillary patients (PB P), with regard to frequency of CD4^+^CD25^high^FOXP3^high^ (A) and CD8^+^CD25^high^FOXP3^high^ (C) cells and to FOXP3 MFI in CD4^+^CD25^high^FOXP3^high^ (B) and CD8^+^CD25^high^FOXP3^high^ (D) cells. Unpaired two-tailed Student t-test, *p*≤0.05 was considered significant. Results are presented as mean with SD.

Interestingly, we observed a well-defined population of CD4^+^/CD8+CD25^high^FOXP3^high^ subsets within CD4^+^ or CD8^+^CD25^high^FOXP3^+^ cells. When we compared patients versus HHC, we found no differences between Treg frequencies (CD4^+^FOXP3^high^, 8.22±4.50 vs. 5.57±4.03%, p = 0.1072 and CD8^+^FOXP3^high^, 3.81±3.35 vs. 2.23±2.68%, p = 0.1718, data not shown). MB patients showed a greater CD4^+^FOXP3^high^ and CD8^+^FOXP3^high^ Treg frequency than HHC, 10.33±5.69 vs. 5.57±4.03% (Figure 3A) and 5.36±4.17 vs. 2.23±2.68% (Figure 3C), respectively.

Furthermore, FOXP3 expression levels in CD4^+^FOXP3^high^ Treg population were greater in patients (1076±140) than in HHC (879.2±193.3), p = 0.0057 (data not shown), especially in MB patients (1098±141.7) (Figure 3B). However, FOXP3 MFI in CD8^+^FOXP3^high^ Treg were not different between patients (1029±213.8) and HHC, (870.5±220.9), p = 0.0651 (data not shown), but MB patients Treg tended to have higher FOXP3 MFI (1088±223.5) than HHC (Figure 3D).

Between PB and MB patients we did not find significant differences in Treg frequency, although it could have tended to be higher in MB patients if a larger number of subjects had been evaluated (Figures 2 and 3).

### Different Profiles in the Frequency of CD4^+^/CD8^+^CD25^+^FOXP3^−^ and CD4^+^/CD8^+^CD25^−^FOXP3^−^ were found between Patients and Contacts

**Figure 4:**
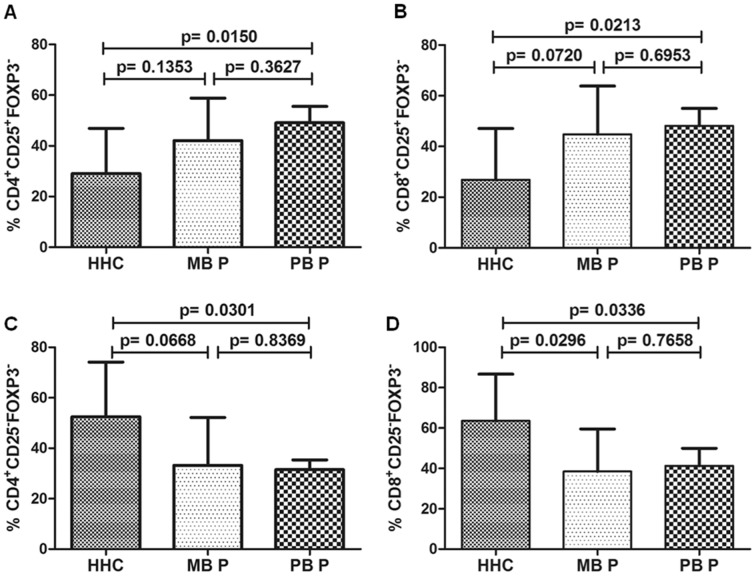
CD4^+^/CD8^+^CD25^+^FOXP3^−^ and CD4^+^/CD8^+^CD25^−^FOXP3^−^ frequency in leprosy patients and contacts. The frequency of CD4^+^/CD8^+^CD25^+^FOXP3^−^ and CD4^+^/CD8^+^CD25^−^FOXP3^−^ cells in CD4^+^ or CD8^+^ lymphocyte gate after 72-hour culture of peripheral blood mononuclear cells with anti-CD3 and anti-CD28 associated with total sonicated fraction of *M. leprae.* Comparison between household contacts (HHC), multibacillary patients (MB P) and paucibacillary patients (PB P), with regard to frequency of CD4^+^CD25^+^FOXP3^−^ (A), CD8^+^CD25^+^FOXP3^−^ (B), CD4^+^CD25^−^FOXP3^−^ (C) and CD8^+^CD25^−^FOXP3^−^ (D) cells. Unpaired two-tailed Student t-test, *p*≤0.05 was considered significant. Results are presented as mean with SD.

We observed that the percentages of CD4^+^ and CD8^+^CD25^+^FOXP3^−^ cells were greater in patients than in HHC (CD4^+^CD25^+^, 45.57±12.65 vs. 29.13±17.79%, p = 0.0107 and CD8^+^CD25^+^, 46.47±13.74 vs. 26.82±20.30%, p = 0.0072, data not shown), especially in PB patients (CD4^+^CD25^+^, 49.07±6.48% and CD8^+^CD25^+^, 48.14±6.93%, figures 4A and B). Furthermore, the percentages of CD4^+^ and CD8^+^CD25^−^FOXP3^−^ cells were lower in patients than in HHC (CD4^+^CD25^−^, 32.37±13.05 vs. 52.51±21.64%, p = 0.0079 and CD8^+^CD25^−^, 39.88±15.45 vs. 63.59±23.15%, p = 0.0046, data not shown). For the CD4^+^CD25^−^FOXP3^−^ population, this difference was evident between PB patients (31.53±3.81%) and HHC (Figure 4C). Regarding CD8^+^CD25^−^FOXP3^−^ cells, both PB (38.45±21.09%) and MB (41.3±8.672%) patients showed a lower frequency of these cells than HHC (Figure 4D).

We also correlated Treg populations with CD4^+^ or CD8^+^CD25^+^FOXP3^−^ cell frequency. There was no correlation in patients, but in HHC there was a positive correlation between all Treg and those subsets’ frequencies (data not shown).

We also evaluated the CD3^+^CD4^+^ and CD3^+^CD8^+^ lymphocyte frequencies, but did not observe differences between patients and HHC or among PB patients, MB patients and HHC (data not shown).

### Treg Frequency is Correlated with the Number of Lesions and Bacillary Index in Patients

**Figure 5:**
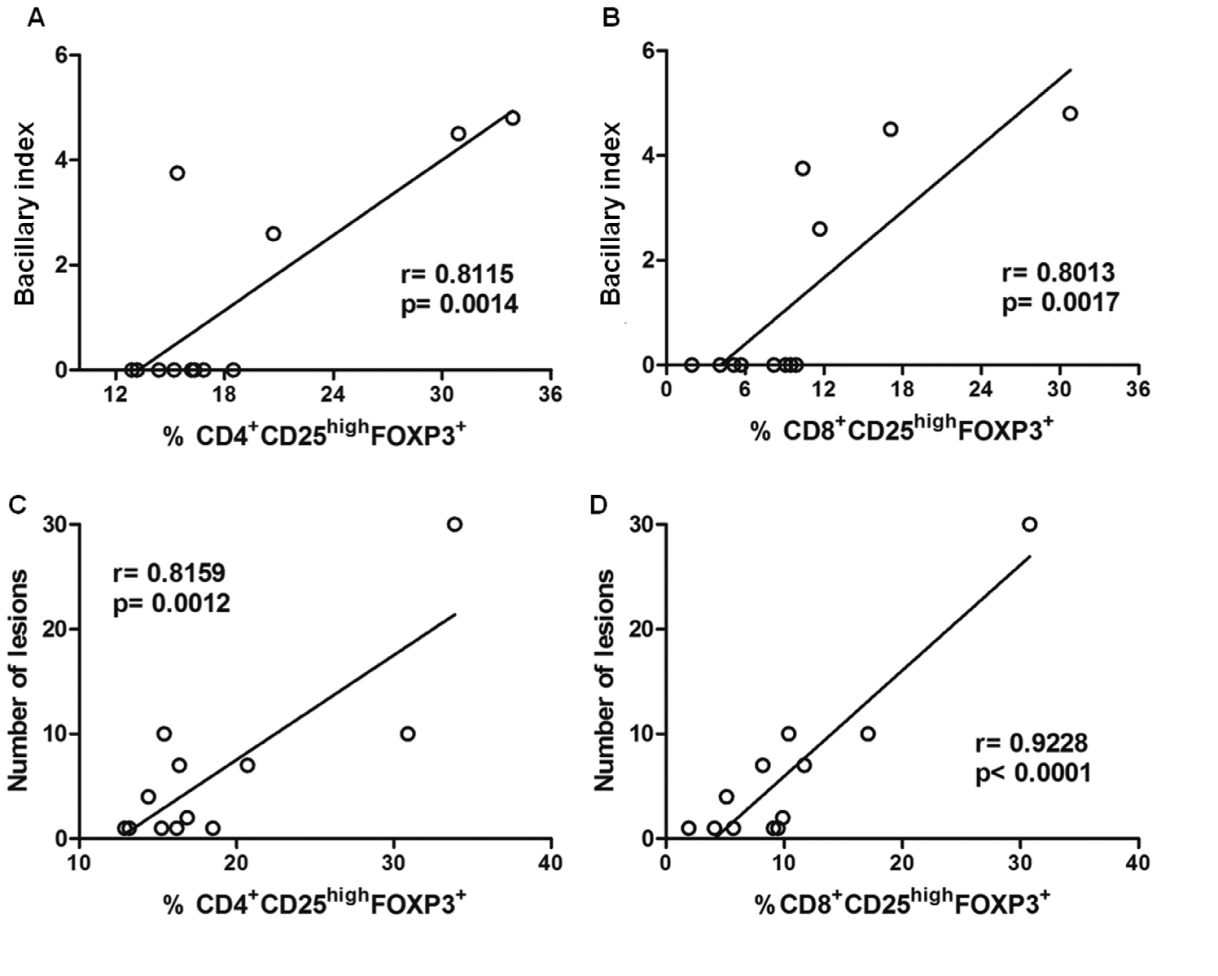
Correlation between CD4^+^/CD8^+^CD25^high^FOXP3^+^ cells and bacillary index and lesion numbers in leprosy patients. The frequency of CD4^+^/CD8^+^CD25^high^FOXP3^+^ cells in CD4^+^ or CD8^+^ lymphocyte gate was obtained after 72-hour culture of peripheral blood mononuclear cells with anti-CD3 and anti-CD28 associated with total sonicated fraction of *M. leprae.* Correlation between bacillary index of leprosy patients and CD4^+^CD25^high^FOXP3 (A) and CD8^+^CD25^high^FOXP3 (B) Treg frequency. Correlation between lesion numbers in leprosy patients and CD4^+^CD25^high^FOXP3 (C) and CD8^+^CD25^high^FOXP3 (D) cell frequency. Pearson test, *p*≤0.05 was considered significant.

There was a strong positive correlation between CD4^+^/CD8^+^CD25^high^FOXP3^+^ Treg frequency and the bacillary index, as illustrated in Figures 5A and 5B, respectively. They were also correlated with lesion numbers (Figures 5C and 5D). CD4^+^/CD8^+^CD25^high^FOXP3^high^ Treg also were positively correlated with the bacillary index (CD4^+^, r = 0.7617; p = 0.004 and CD8^+^, r = 0.7654; p = 0.0037, data not shown) and also with lesion numbers (CD4^+^, r = 0.7899; p = 0.0022 and CD8^+^, r = 0.93; p<0.0001, data not shown).

We did not find a correlation between CD4^+^ or CD8^+^CD25^+^FOXP3^−^ cell frequency and those variables, lesion numbers and bacillary index, which may indicate that these subsets are not directly responsible for the inflammatory state in MB patients.

## Discussion

This is the first work investigating CD4^+^ and CD8^+^ Treg in leprosy patients and HHC under 15 years old. Although it has been many years since the existence of a suppressor population was described in patients with leprosy [20], [30], there is still controversy over its role in the emergence of the disease and the different clinical presentations.

Macrophage activation is an important step for *M. leprae* infection control. Progression to multibacillary leprosy is characterized by a state of T cell hyporesponsiveness, with consequent loss of the macrophages’ microbicidal capacity [31]. In this study, we showed that in individuals under 15 years old, the numbers of CD4^+^CD25^high^FOXP3^+^ cells and their activated fraction, CD4^+^CD25^high^FOXP3^high^ cells, were higher in MB leprosy patients compared to HHC.

Our results are similar to those of Palermo et al., who found in lepromatous patients an increased frequency of CD4^+^CD25^+^FOXP3^+^ population after culture with *M. leprae* cell wall antigens (MLCwA) and increased expression of negative regulatory molecules in lesion biopsies [32]. However, the population analyzed included activated cells without regulatory function, which can express CD25^+^ and FOXP3^+^ at lower levels than Treg [14], [33].

Atia et al. detected a greater frequency of CD4^+^CD25^high^FOXP3^+^ population in *ex-vivo* samples of adult patients with the tuberculoid form of leprosy when compared to controls [34]. However, the gating strategy used by those authors to define CD25^high^ differed from ours, and they did not examine other Treg populations, such as CD8^+^ Treg and CD25^high^FOXP3^high^ Treg, which can explain the discrepant results at least in part.

Massone et al. evaluated Treg in biopsies of leprosy patients with different clinical forms and demonstrated an increased frequency of FOXP3^+^ cells only in patients with reversal reaction [35]. In that study, the authors used only FOXP3 as a marker for Treg identification in a small number of patients, which made it difficult to obtain significant results with non-reaction lepromatous patients.

CD8^+^ T cells, called suppressors, were initially identified in the lesions of patients with lepromatous leprosy, and were found to be capable of suppressing the response of PBMC to concanavalin A stimulation [21]. Clones of CD8^+^ T cells isolated from peripheral blood of patients with lepromatous leprosy inhibited the activation of PBMC by *M. leprae* antigens *in vitro* [22]. In this study, we showed for the first time an increased frequency of CD8^+^CD25^high^FOXP3^+^ and CD8^+^CD25^high^FOXP3^high^ cells in MB patients compared to HHC. We believe that these cells may be those with suppressive capacity observed in these previous studies.

Although we did not use other activation markers beyond CD25, we believe that CD25^+^FOXP3^−^ cells are activated/effector T cells in their majority, as demonstrated by Miyara et al. Those authors demonstrated that the fraction CD25^+^FOXP3^−^, isolated from human peripheral blood, did not have suppressor function and instead enhanced the responder proliferation [15]. The higher CD4^+^/CD8^+^CD25^+^FOXP3^−^ cell frequency in PB patients observed in our study may reflect immune activation and cell-mediated immune response in those patients, as described by Scollard et al. [7]. We believe that CD4^+^/CD8^+^CD25^+^FOXP3^−^ cells in HHC may counterregulate Treg subsets, as suggested by the positive correlation between them, maintaining homeostasis and preventing the disease.

In addition, we observed a greater FOXP3 expression (MFI) by the CD4^+^ and CD8^+^ Treg in MB patients than in HHC. Although the suppressive potential of Treg and the relation with FOXP3 expression has not been investigated in leprosy, the FOXP3 expression was more related with Treg suppressive potential than the Treg frequency in mice [36]. In humans, FOXP3 MFI also was related with suppression mediated by Treg in the autoimmune disease model [37].

It is not clear how Treg expand in *M. leprae* infection, but it is known that pathogens can modulate the immune system in favor of their survival [10]. It is speculated that lipids of mycobacterial origin may have a regulatory function. Mannose-capped lipoarabinomannan, a glycolipid expressed by *M. tuberculosis* and *M. leprae* [38], is able to expand Treg cells in healthy tuberculin reactors [39]. Phenolic glycolipid-1 (PGL-1), a specific antigen of *M. leprae*, may also have immunosuppressive properties [7]. Furthermore, in skin biopsies, lepromatous leprosy patients showed a higher number of indoleamine 2,3-dioxygenase (IDO)-positive cells than borderline tuberculoid patients, and *M. leprae* increased the IDO expression in PBMC of healthy controls [40]. IDO is an enzyme that can suppress effector T cells by degrading tryptophan and by inducing Treg cells [41]. These factors suggest that components of *M. leprae* can modulate the immune response through induction of Treg, which agrees with our finding of a positive correlation between Treg frequency and bacillary index on the one hand and the number of lesions in patients on the other.

One limitation of our study was the small sample size for each clinical form. A larger number of MB and PB patients would help show differences between these groups and might corroborate that PB patients have similar Treg frequency as contacts. Another important question is that PBMC expansion using non-specific stimuli could reflect Treg which are not involved directly in leprosy immunopathogenesis. However, stimulation with anti-CD3 and anti-CD28 is widely used for Treg expansion in the literature [42]–[44]. Moreover our finding of a positive correlation between Treg and patients’ lesion number and bacillary index suggests that the Treg frequency found is linked to *M. leprae* infection.

To verify the functional activity of Treg in leprosy and their main suppression mechanisms, it is necessary to conduct further research with adult patients, since those assays need a larger blood volume.

Our results allow us to speculate that infection by *M. leprae* can modulate the immune response, with the induction of Treg, for creation of a favorable environment for its survival. A follow-up study of the contacts to monitor the occurrence of illness and changes in Treg frequency is important to define these cells as a biomarker of leprosy.
